# Retrospective analysis of episiotomy prevalence

**DOI:** 10.4274/jtgga.2016.0238

**Published:** 2017-12-15

**Authors:** Bahtışen Kartal, Aynur Kızılırmak, Pelin Calpbinici, Gökçe Demir

**Affiliations:** 1 Department of Nursing, Gaziosmanpaşa University Faculty of Health Sciences, Tokat, Turkey; 2 Department of Nursing, Nevşehir Hacı Bektaş Veli University Semra and Vefa Küçük School of Health, Nevşehir, Turkey; 3 Nevşehir Hacı Bektaş Veli University Semra and Vefa Küçük School of Health, Nevşehir, Turkey; 4 Department of Nursing, Ahi Evran University School of Health, Kırşehir, Turkey

**Keywords:** Episiotomy, prevalence, vaginal deliveries

## Abstract

**Objective:**

This study was performed to determine the rate of episiotomy.

**Material and Methods:**

This retrospective was conducted in 3 state hospitals located in 3 cities in the Central Anatolia region of Turkey. Ethics committee approval was received for this study. Also, institutional permissions from the institutions where the study was conducted were obtained before the study. The sample of the study consisted of 8587 women. The data of the study were collected by analyzing birth records in archive records.

**Results:**

The average age of the women was 26.16±5.9 years, the average number of deliveries was 2.19±1.2, and 52.0% of the women who gave birth via vaginal delivery underwent episiotomy. The rate of episiotomy was found to be 93.3% in primipara women and 30.2% in multipara women. It was determined that neonatal weight did not affect the episiotomy rate, and that neonatal height was higher in deliveries with episiotomy and suture. Also, it was determined that as the age and parity of the women decreased, the rate of episiotomy increased.

**Conclusion:**

The rate of episiotomy was observed to be high, especially in primipara women.

## INTRODUCTION

Episiotomy is a surgical incision applied to the bulbocavernosus muscle in the second phase of labor in order to make the delivery easier by enlarging the vaginal opening, to protect the tonus of the perineum, to prevent undesired vaginal fissures, and to enable easy, fast and safe delivery of the head of the fetus (1).

Surgical opening of the perineum was suggested for the first time in 1714 in order to prevent serious tears of the perineum (2). A significant increase in episiotomy rates was observed around the world (3). Despite being one of the most frequently administered surgical procedures in the world, the efficacy of episiotomy was introduced without strong scientific evidence (2). The World Health Organization (WHO) suggested that episiotomy should not be administered as routine practice (4), and in a bulletin published by the American College of Obstetricians and Gynecologists, episiotomy was reported to be restricted (5). Despite these suggestions, prevalence of episiotomy varies significantly between countries (6). The rate of episiotomy varies between 9.7% (the lowest) (Sweden) and 100% (the highest) (Taiwan) in both primipara and multipara women (7).

Episiotomy is suggested to be administered in conditions such as complicated vaginal deliveries (breech, shoulder dystocia, forceps, vacuum), incision-related scars in the genital area, poorly healed or 4th degree tears, and fetal distress (8). There are different opinions about the applicability of episiotomy in addition to protecting maternal and infant health. While opinions about episiotomy’s increasing Apgar score of the baby or decreasing perinatal asphyxia by shortening the second phase of delivery are not definite, there are also views that it does not prevent, or even increases, defects in the perineum (9). Also, in a comparison of limited use of episiotomy and routine episiotomy in deliveries without any complication, the WHO reported that episiotomy decreased posterior perineal trauma risk and prenatal trauma repair need, and that there was no difference between the two groups in terms of risks of vaginal and perineal trauma, pain, dyspareunia, and urinary incontinence (10).

It is indicated that routinely administered episiotomy causes postpartum early perineal complications and higher perineal pain scores (11-13), urinary inconsistencey is higher in the postpartum 3rd month in women undergoing episiotomy (13), and the amount of blood loss is higher in the delivery (14). In another study, it was stated that with a decrease of episiotomy administration, anal sphincter lacerations decreased in vaginal deliveries (15).

The rate of perineal trauma is indicated to be high in countries where episiotomy is frequently administered (16, 17,18). Moreover, perineal trauma caused due to episiotomy can affect the sexuality and self-confidence of women (19,20), and lead to perineal pain and infections (21,22). There are also studies emphasizing that episiotomy has a protective role against the formation of 3rd degree tears (14,23,24,25).

The study was conducted to retrospectively analyze the prevalence of episiotomy in vaginal deliveries in 3 state hospitals located in the Central Anatolia region of Turkey.

## MATERIAL AND METHODS

### Study design and participants

This retrospective study was conducted in state hospitals in 3 cities in the Central Anatolia region of Turkey. The records of 8649 women who gave birth between January 1st, and December 31st, 2013, were examined retrospectively. The data of 62 women were excluded due to a lack of information and 8587 women were included in the study. The data of the study were collected by examining birth registrations from archive records. The birth registrations involved information concerning the ages of the women, delivery methods, number of births, and the height and weight of the infants.

### Ethical aspect of the study

Ethics committee approval was received for this study. Also, institutional permissions from the institutions where the study was conducted were obtained before the study.

### Statistical analysis

The Statistical Package for Social Sciences (SPSS) 20 package programme was used to assess the data. Categorical measurements are given as number and percentage, and numerical measurements are given as mean and standard deviation. The chi-square and ANOVA test were used. Statistical significance was accepted as p<0.05.

## RESULTS

It was determined that average age of the women was 26.16±5.9 years, the average number of births was 2.19±1.2, 34.6% were primipara, 76.4% of all women had vaginal deliveries, and 52.0% of the women who had vaginal deliveries gave birth with episiotomy. Also, 99.2% of the deliveries were live births (Table 1).

The rate of episiotomy was determined as 93.3% in primipara women and as 30.2% in multipara women. The rate of suture delivery without episiotomy was 0.6% in primipara women, whereas this rate was 7.4% in multipara women. In the statistical analysis, a significant difference between the groups was determined (p<0.05) (Table 2).

It was determined that neonatal weight did not affect the episiotomy rate, and neonatal height was higher in deliveries with episiotomy and suture. Moreover, as the age of the women decreased, the episiotomy rate increased. The episiotomy rate of women with a low number of births was high and the difference between the groups was statistically significant (p<0.05) (Table 3).

## DISCUSSION

The WHO reported that episiotomy should be restricted in deliveries without complications. In the same study, it was also stated that restrictive episiotomy was more advantageous compared with routine episiotomy, and there was less posterior perineal trauma, and fewer sutures and complications in restrictive episiotomy (2).

In addition to the WHO and other authorities, patients for whom episiotomy should be administered are clearly defined in the safe motherhood module published by the General Directorate of Maternal and Infant Health and Family Planning and used in in-service training of personnel. Episiotomy is suggested in order to step up the delivery in cases with fetal distress, in order to prevent intracranial hemorrhage with forceps, vacuum applications, premature or breech delivery, and in cases where excertion of the mother’s strength during delivery should be prevented (i.e. cardiac failure), and if there is a risk of 3rd degree perineum tears (especially when 3rd degree tears occurred during a previous delivery) (26).

In the present study, it was determined that more than half of the women (52.0%) having vaginal deliveries underwent episiotomy, and 93.3% of the primipara women and 30.2% of the multipara women received episiotomy. The suture delivery rate was determined to be higher in multipara women.

In a study conducted by Çalışkan et al. (27) (2003), it was reported that the episiotomy rate was 74.2%. In another study conducted in Turkey by Karaçam et al. (12), it was reported that episiotomy was performed in 64% of vaginal deliveries (95% of first deliveries, 48% of second deliveries, 12% of third and subsequent deliveries). In another Turkish study, episiotomy was reported to be administered in 92% of primipara women and 72% of multipara women (28).

In some countries, the episiotomy rate has decreased over the years. The episiotomy rate was 60.9% in all vaginal deliveries in 1979 in the United States of America, but the rate decreased to 24.5% in 2004; (15) in a study conducted in Thomas Jefferson University Hospital, the episiotomy rate decreased from 69.6% in 1983 to 19.4% in 2000 (29), and in a study conducted in Hong Kong, the rate decreased from 73% in 2003 to 27% in 2008 (30). However, the ideal rate of episiotomy is still not clear (15). There are differences between episiotomy rates depending on the countries. In a study conducted in primipara women in Nigeria, the rate of episiotomy was determined as 62.1% (6). In contrast, the rate of episiotomy was 40.6% in primipara women in a study conducted in Italy (31). Trinh et al. (32) (2013) evaluated the rate of episiotomy among women born in Vietnam and Australia between 2001 and 2010. In Australia, they found that the episiotomy rate was 27% in Australian-born primipara women, and 48% in Vietnamese-born women. In a study conducted in Oman, the rate of episiotomy was 66% (33).

Perineal trauma is described as damage that occurs in the genital region or due to a surgical incision or episiotomy during delivery (20,34). Even though there are a number of studies indicating that episiotomy is defined as a cause of birth trauma, as well as disadvantages of its administration, the episiotomy rate was high in the present study, as it is in developing countries. Episiotomy is administered in almost all primipara women regardless of the presence/absence of complications with delivery, it is almost a routine administration for primipara women. In Turkey, deliveries performed at hospitals are performed in the lithotomy position and practices providing flexibility to the perineum are applied in very few clinics. Episiotomy administration procedures should be adapted to all healthcare personnel who assist delivery through in-service training and the necessity of avoiding routine administration should be emphasized. In addition, increasing alternative practices such as massage and restricting episiotomy in vaginal deliveries will enable a decrease in the episiotomy rate.

## Figures and Tables

**Table 1:**
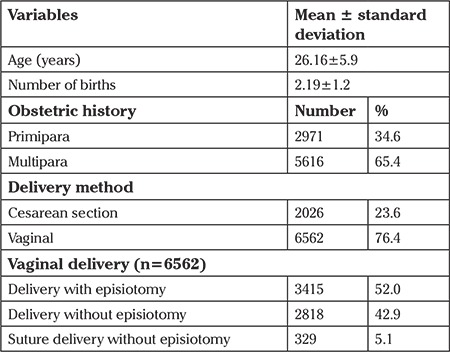
Distribution of the women based on demographic and obstetric characteristics

**Table 2:**
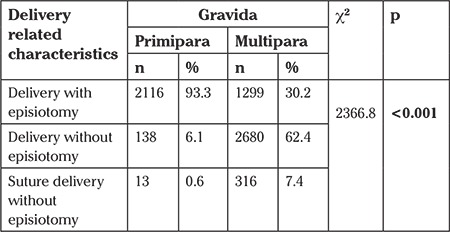
Distribution of vaginal delivery-related characteristics of the women in terms of gravida number

**Table 3:**
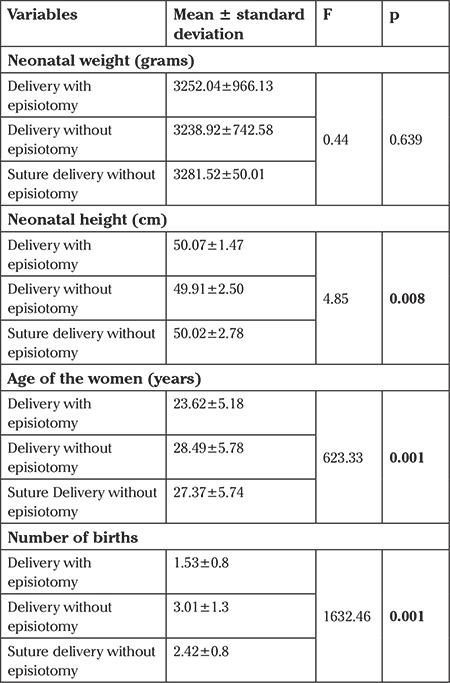
Distribution of vaginal delivery characteristics in terms of different variables
